# Protective Effects of Human Dermal Fibroblast-Derived Extracellular Vesicles on WI-38 Lung Fibroblasts Under Different Stress Conditions

**DOI:** 10.3390/cimb48101052

**Published:** 2026-10-12

**Authors:** Dilek Bahar

**Affiliations:** Genome and Stem Cell Centre (GENKOK), Erciyes University, 38280 Kayseri, Turkey; dilekbahar@erciyes.edu.tr

**Keywords:** extracellular vesicle, Wl-38, oxidative stress, chemical hypoxia, apoptosis, miR-21-5p

## Abstract

In lung fibroblasts, cellular stress can disrupt tissue homeostasis through the accumulation of reactive oxygen species (ROS), loss of membrane integrity, and apoptotic cell death. In this study, the protective effects of extracellular vesicles (EV) isolated from human fibroblasts by ultracentrifugation were investigated in normal human lung fibroblast Wl-38 cells exposed to three stress models induced by heat, H_2_O_2_, and CoCl_2_. Cells were subjected to heat stress at 42 °C for 1 h, 40 µM H_2_O_2_, and 500 µM CoCl_2_; extracellular vesicles treatment was administered 1 h after the onset of stress, and analyses were performed 24 h later. The extracellular vesicle dose–response was evaluated using the MTT assay; ROS, apoptosis, propidium-iodide (PI) uptake, and miR-21-5p expression were assessed using the relevant methods. All three stress models significantly increased ROS and total apoptosis. Extracellular vesicle treatment reduced ROS levels; reduced total apoptosis from 55.06% to 33.40% under heat stress, from 55.16% to 43.40% under H_2_O_2_ stress, and from 54.43% to 32.40% under chemical hypoxia. PI values also decreased significantly in all three models. A significant decrease in miR-21-5p expression was observed in the exosome-treated groups compared to the stress groups. The results suggest that fibroblast-derived extracellular vesicles may exhibit a common cytoprotective phenotype that reduces to various stress stimuli.

## 1. Introduction

Global warming, air pollution, and exposure to environmental chemicals are among the major environmentally derived stressors that affect human health through both direct and indirect mechanisms. Although these factors have distinct characteristics, they can converge on common stress pathways at the cellular level, such as increased reactive oxygen species, disruption of protein homeostasis, and triggering of cell death mechanisms [1,2]. Lung tissue is particularly sensitive to environmental stressors because it is in constant contact with the external environment over a large surface area. Lung fibroblasts, in turn, play a central role in the production and remodelling of the extracellular matrix, the maintenance of tissue architecture, and the execution of post-injury repair. However, environmental stresses—such as oxidants, temperature changes, and hypoxia-associated signals—can disrupt the redox balance of fibroblasts, leading to reduced proliferation, membrane damage, senescence, or apoptotic cell death [3,4].

While heat stress affects protein folding, mitochondrial function, and antioxidant defence, H_2_O_2_ is a common in vitro stressor that directly induces oxidative load. CoCl_2_, on the other hand, mimics HIF-1α stabilisation and induces a chemical hypoxia-like response; it is important to evaluate the resulting cytotoxicity alongside the hypoxia signal [5,6]. Hypoxia is an important feature of several pulmonary pathologies and contributes to cellular dysfunction. Therefore, CoCl_2_-induced chemical hypoxia was included as a relevant in vitro stress model [6].

Extracellular vesicles (EVs) are intercellular communication vehicles that can modulate the stress responses of recipient cells through the proteins, lipids, and regulatory nucleic acids they carry. These properties make extracellular vesicles potential protective biological agents that reduce cellular damage and support tissue homeostasis under various stress conditions. Current MISEV2023 guidelines recommend using the more general term “EV” when the biogenesis origin of vesicle subtypes is not directly demonstrated, and call for detailed reporting of isolation, characterisation, and functional experiments [7]. For this reason, the term “EV” has been used in our study.

However, extracellular vesicles have emerged as a significant link between environmental pollutants and their adverse health effects [8]. Emerging research indicates that environmental pollutants can significantly influence exosome biogenesis, release, and cargo composition, which in turn may potentially lead to cellular stress and disease progression [9].

Cellular stress represents an important contributor to tissue dysfunction and cellular damage, highlighting the need for strategies capable of limiting stress-induced injury. Although EVs have attracted considerable attention because of their regenerative and cytoprotective potential, research has predominantly focused on MSC-derived EVs, whereas the protective effects of fibroblast-derived EVs remain comparatively less explored. In particular, it remains unclear whether fibroblast-derived EVs can provide cytoprotection across distinct forms of cellular stress.

Accordingly, this study aimed to investigate the protective effects of EVs derived from human fibroblasts on normal lung fibroblasts under conditions of heat stress, oxidative stress induced by H_2_O_2_, and chemical hypoxia induced by CoCl_2_. By evaluating the effects of EV administration on cell viability, ROS production, membrane integrity, apoptosis, and miR-21-5p expression, the study aimed to identify common or model-specific cellular responses

## 2. Materials and Methods

### 2.1. Cell Culture

The normal human lung fibroblast cell line Wl-38 (ATCC CCL-75, Manassas, VA, USA) was used for this procedure. The cells were cultured in complete medium containing 10% fetal bovine serum, 1% penicillin–streptomycin, and L-glutamine (EMEM; Gibco, Waltham, MA, USA) at 37 °C in a 5% CO_2_ humidified atmosphere. WI-38 normal human lung fibroblasts (ATCC CCL-75, Manassas, VA, USA) were used as the recipient cell model for the stress experiments, whereas normal human adult dermal fibroblasts (ATCC PCS-201-012, Manassas, VA, USA) were used exclusively as the source cells for EV production.

### 2.2. Isolation and Characterisation of Fibroblast-Derived EVs

Human fibroblast cells (ATCC PCS-201-012, Manassas, VA, USA) were used for the production of extracellular vesicles. These cells were cultured in a complete medium containing DMEM (4.500 g/L D-glucose, Cat. No. 41966-029 Gibco, Waltham, MA, USA), 10% fetal bovine serum, 1% penicillin–streptomycin, and L-glutamine under conditions of 37 °C, 5% CO_2_, and a humidified atmosphere. To collect extracellular vesicles, the cells were cultured overnight in FBS-free medium, to minimise contamination of EV preparations with serum-derived vesicles, bovine proteins, and other serum components, the cells were washed twice and subsequently maintained in FBS-free medium for approximately 24 h before collection of the conditioned medium for EV isolation. The following day, the medium was harvested and subjected to successive centrifugation steps (300× *g* for 30 min) to remove the medium, cells, and cellular debris. The resulting supernatant was filtered through a 0.22 µm filter and centrifuged twice by ultracentrifugation (120,000× *g*, 90 min, HIMAC, CP100NX, P55STN2, Eppendorf Himac Technologies Co., Ltd., Ibaraki, Japan) to isolate extracellular vesicles, then resuspended in DPBS. The total protein concentration of the preparation was measured by microBCA (ThermoScientific, Waltham, MA, USA) and was 500 µg/mL. The preparations were stored at −80 °C until use.

Extracellular characterisation procedures were then performed. Scanning electron microscopy (SEM) analysis was conducted for morphological characterisation. 30 μL of exosome samples were dropped onto a clean microscope slide and dried in a sterile laminar flow hood; they were then coated with Au-Pa. The samples were subsequently imaged. Nanoparticle analysis (NTA) was performed to determine the quantity and size distribution of the extracellular vesicles. The samples were diluted 1:1000 with ddH_2_O and analysed using an NTA instrument (Malvern Panalytical, Malvern, UK). Immediately prior to NTA, EV samples were diluted in distilled water to achieve a particle concentration suitable for analysis, following the measurement protocol recommended for the instrument. Distilled water was used only as the diluent for NTA measurement and was not used during EV isolation or storage. Surface markers of the extracellular vesicles were characterised via biochemical analysis using flow cytometry. For this purpose, latex beads (Thermo Fisher, Waltham, MA, USA) were added to a 50 µg/mL sample and the mixture was incubated overnight at +4 °C. Subsequently, 2 M glycine was added, and the mixture was incubated at +4 °C for 30 min. The samples were then centrifuged at 20,000× *g* for 10 min. The pellet was resuspended in dPBS, and anti-CD63 and anti-CD81 antibodies (Biolegend, San Diego, CA, USA) were added to the samples, which were then incubated in the dark at room temperature for 1 h. Following washing, the samples were analysed by flow cytometry (FACS Aria III, BD Biosciences, San Jose, CA, USA). FlowJo software (version 10, BD Life Sciences, Ashland, OR, USA) was used to perform the flow cytometry data analysis [10].

### 2.3. Preparation of EV Doses

In the MTT dose–response experiments, 100, 50, 25, 12.5, and 6.25 µL were used from a 500 µg/mL stock solution. Since the final well volume was 200 µL, the corresponding final protein-equivalent concentrations were 250, 125, 62.5, 31.25, and 15.625 µg/mL, respectively. In subsequent analyses, the 100 µL (250 µg/mL) dose, which produced the most pronounced protective response according to the MTT results, was used.

### 2.4. Stress Models and Experimental Groups

Preliminary optimisation experiments were conducted to determine the stress conditions for the study. WI-38 cells were exposed to different concentrations of H_2_O_2_ and CoCl_2_ and to various heat stress conditions. Following the treatments, cell viability was assessed using the MTT assay. Based on the obtained dose–response results, experimental concentrations were determined for H_2_O_2_ and CoCl_2_ that reduced cell viability by approximately 50% compared to the control group. For heat stress, the temperature and exposure time that caused an approximately 50% reduction in cell viability were used as the basis. These determined conditions were used as standard stress conditions in subsequent EV application experiments. For heat stress, the cells were incubated at 42 °C for 1 h. For oxidative stress, a diluted working solution prepared from 3% H_2_O_2_ (Boster Bio, AR1108 Pleasanton, CA, USA) was used to achieve a final H_2_O_2_ concentration of 40 µM in the culture medium. The chemical hypoxia model was established using 500 µM CoCl_2_. EV was added to the treatment groups 1 h after the onset of each stress stimulus. H_2_O_2_ and CoCl_2_ were not removed from the medium; analyses were performed 24 h after EV treatment. The groups were organized as follows: control, heat stress, heat stress + EV, H_2_O_2_ stress, H_2_O_2_ stress + EV, chemical hypoxia with CoCl_2_, and chemical hypoxia + EV.

### 2.5. MTT Viability Assay

Cell viability was assessed using the standard MTT reduction assay. Wl-38 cells were seeded into 96-well plates at a density of 5 × 10^3^ cells per well. Twenty-four hours after the experimental groups were established, a final concentration of 0.5 mg/mL MTT solution was added to each well, and the plates were incubated for approximately 3 h. The resulting formazan crystals were dissolved in DMSO, and absorbance was measured at 560 nm (Promega Glomax ELISA Reader, Promega Corporation, Madison, WI, USA). Results are presented as raw absorbance/normalised viability.

### 2.6. ROS Analysis

Intracellular oxidative stress was assessed using the Muse^®^ Oxidative Stress Kit (Luminex Corporation, Austin, TX, USA) according to the manufacturer’s protocol. After treatment, cells were harvested, adjusted to the appropriate cell density, incubated with the kit reagent, and analysed on the Muse^®^ Cell Analyser (Luminex Corporation, Austin, TX, USA). Results were recorded as the percentage of ROS-positive cells and the ROS level generated by the kit.

### 2.7. Apoptosis and Membrane Integrity

Apoptosis was analysed using the Muse^®^ Annexin V & Dead Cell Kit (Luminex Corporation, Austin, TX, USA) according to the manufacturer’s protocol. Live, early apoptotic, and late apoptotic/dead cell fractions were determined using the Muse^®^ Cell Analyser (Luminex Corporation, Austin, TX, USA), and total apoptosis was calculated as the sum of the early and late apoptotic fractions. Membrane integrity was also assessed by propidium iodide (PI) uptake. Since PI cannot cross an intact cell membrane, an increased PI signal was associated with late apoptotic or necrotic cells exhibiting a loss of membrane integrity. The PI parameter was reported as (percentage of PI-positive cells/mean Hoechst fluorescence intensity).

### 2.8. RNA Isolation and miR-21-5p RT-qPCR

Wl-38 cells were harvested 24 h after treatment and incubated with 1000 µL of QIAzol lysis buffer (QIAGEN, Hilden, Germany) at room temperature for 5 min. Chloroform (200 µL) was added to the samples, which were vortexed for 15 s and centrifuged at 12,000× *g* for 15 min. The aqueous phase was transferred to a new tube and incubated with 500 µL isopropanol at −20 °C for 10 min. The samples were centrifuged at 12,000× *g* for 10 min at 4 °C; the pellet was washed with 75% ethanol (7500× *g* for 5 min at 4 °C), dried, and dissolved in 30 µL of nuclease-free water. RNA concentration was measured in ng/µL using a NanoDrop (Thermo Fisher Scientific, Waltham, MA, USA) instrument.

cDNA synthesis was performed in a total volume of 10 µL using the miRCURY LNA RT Kit (QIAGEN, Hilden, Germany): 2 µL of 5× reaction buffer, 1 µL of 10× miRCURY RT enzyme mixture, 2 µL of total RNA, and 5 µL of nuclease-free water. The reaction was run on a SensoQuest Labcycler (SensoQuest GmbH, Göttingen, Germany). qPCR was performed on a Roche LightCycler 480 (Roche Diagnostics, Rotkreuz, Switzerland) using the miRCURY LNA SYBR Green PCR Kit (QIAGEN, Hilden, Germany) in a total volume of 10 µL (5 µL 2× master mix, 1 µL primer mixture, 3 µL cDNA, and 1 µL water). miR-21-5p expression was normalized to U6, and relative expression was calculated using the 2^−ΔΔCt^ method (hsa-miR-21-5p, #339306miRCURY, QIAGEN, Hilden, Germany).

### 2.9. Statistical Analysis

Data from three independent biological experiments are presented as mean. A one-way ANOVA test was performed. In the PI analysis, paired stress–EV comparisons were evaluated using a two-way paired *t*-test. A *p*-value < 0.05 was considered statistically significant. Analyses were performed using R software (version 4.5; R Foundation for Statistical Computing, Vienna, Austria).

## 3. Results

**Figure 1:**
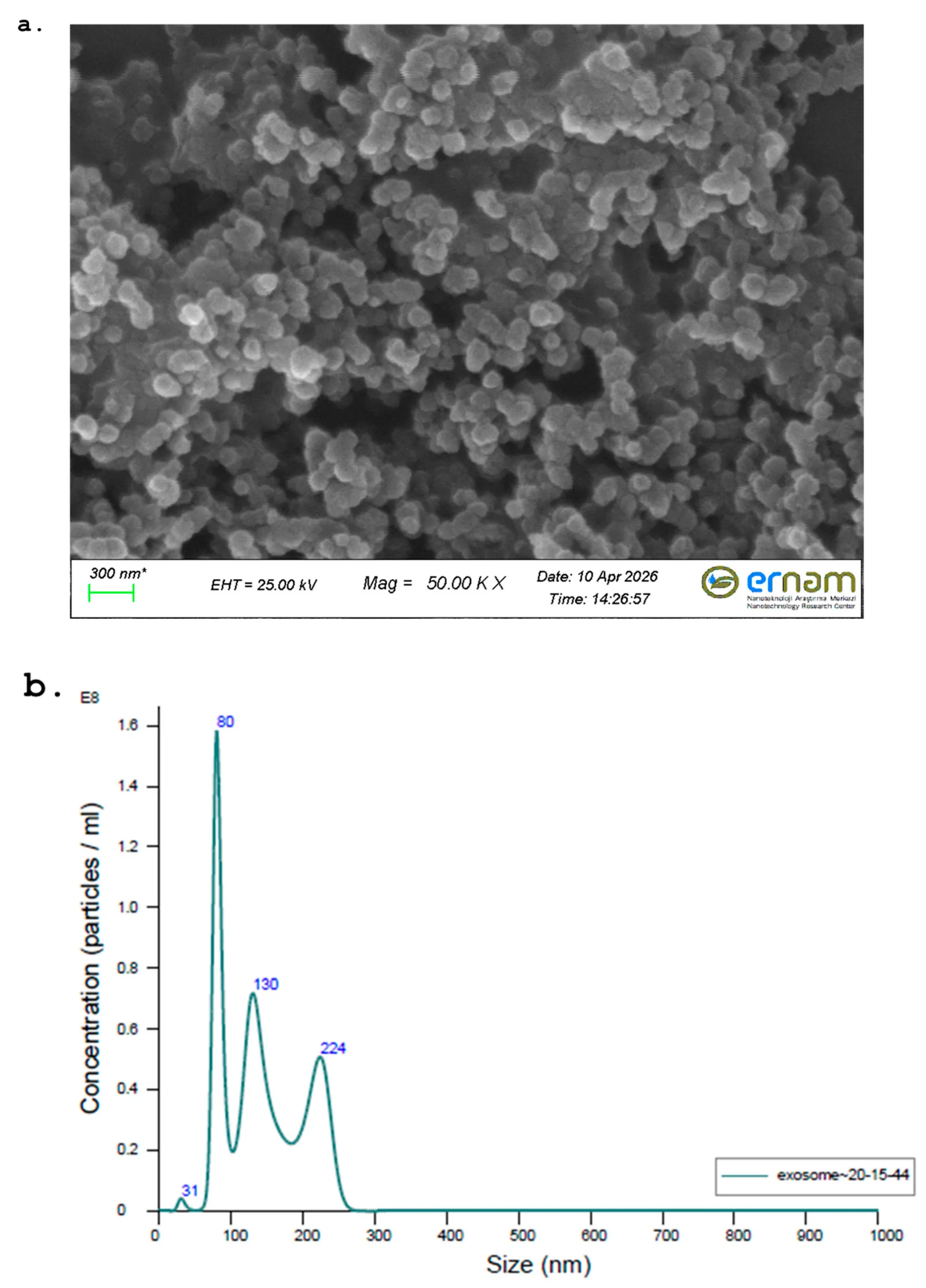

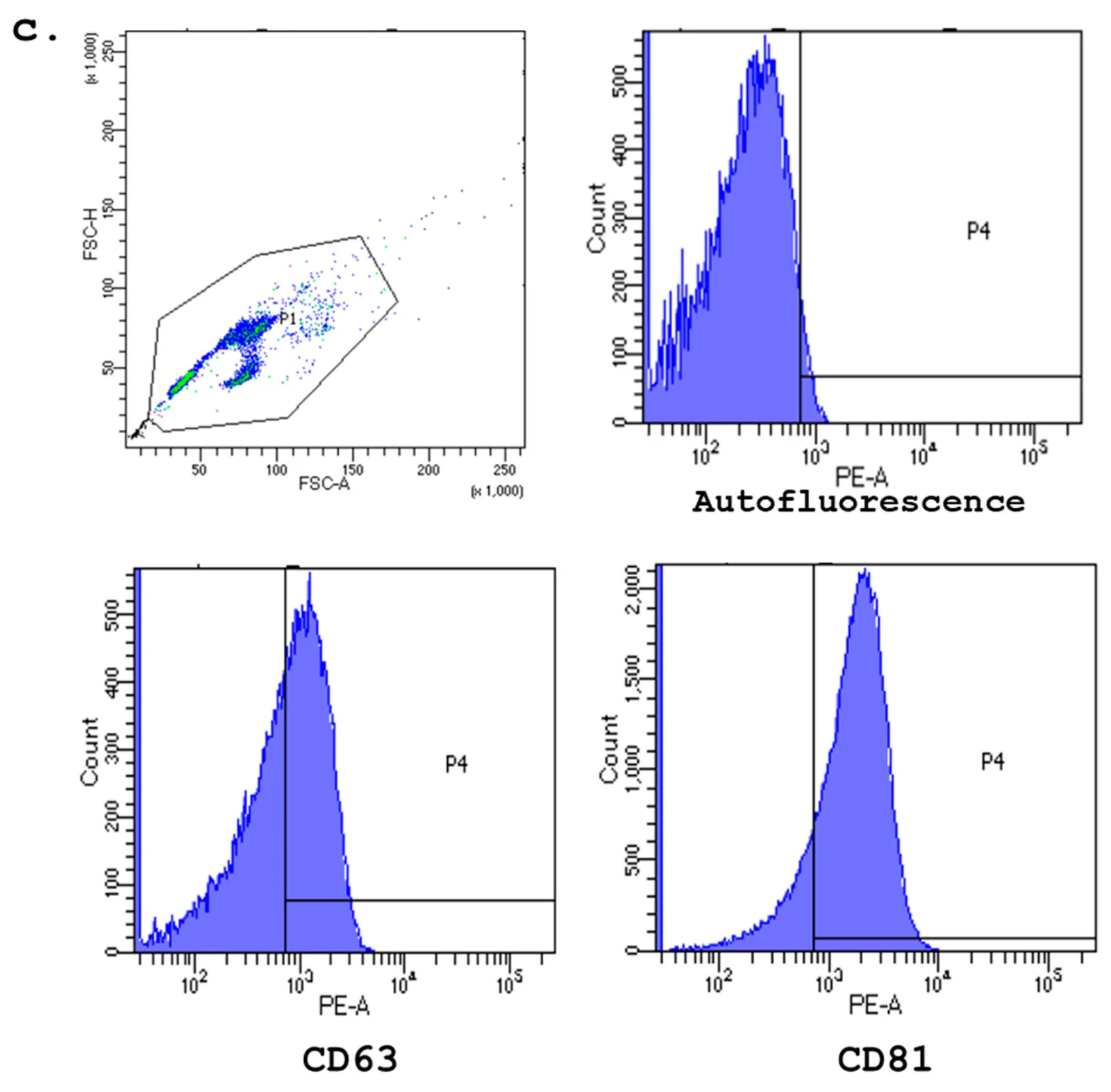
Results of characterisation tests for extracellular vesicles. (**a**) SEM images of the samples, Mag = 5000 KX, the scale bar in the SEM image represents 300 nm.; (**b**) NTA plots of the samples, showing that the samples are distributed between 31 and 224 nm; (**c**) surface markers of the samples; the isolated samples are positive for CD63 and CD81. The scale bar represents 300 nm, and the asterisk is part of the original SEM image annotation.

The isolated vesicles were characterized using NTA, TEM, and EV-associated CD9- and CD63-positive markers (Figure 1). The results demonstrated that the isolated extracellular vesicles exhibited characteristics similar to those of EVs.

### 3.1. EVs Increased Metabolic Vitality in Three Stress Models

**Figure 2:**
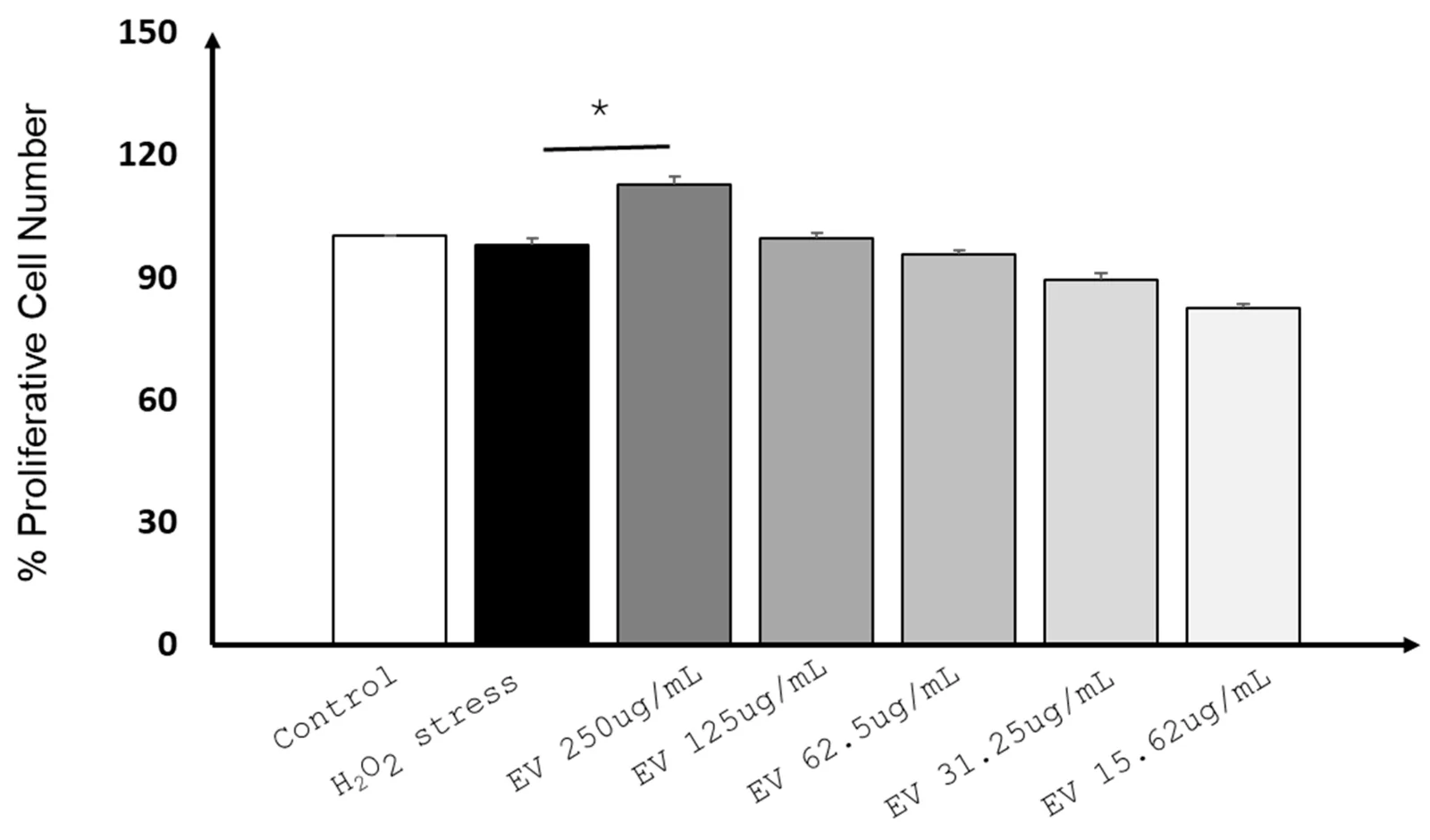
Effect of EV doses on the MTT response of Wl-38 cells under H_2_O_2_-induced oxidative stress (*n* = 3), * *p* < 0.05.

**Figure 3:**
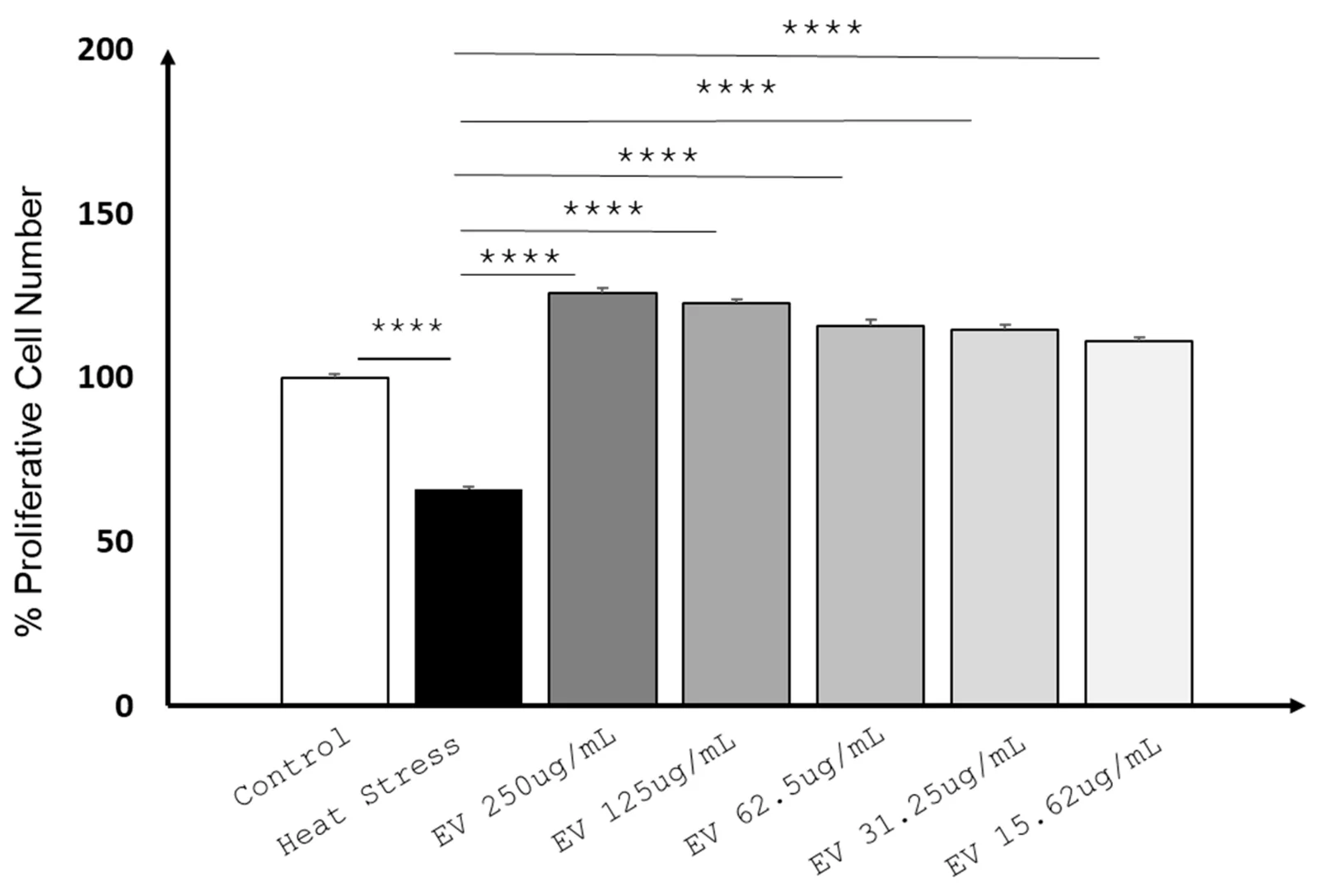
Effect of different EV doses on the MTT response under heat stress (*n* = 3), **** *p* < 0.0001.

**Figure 4:**
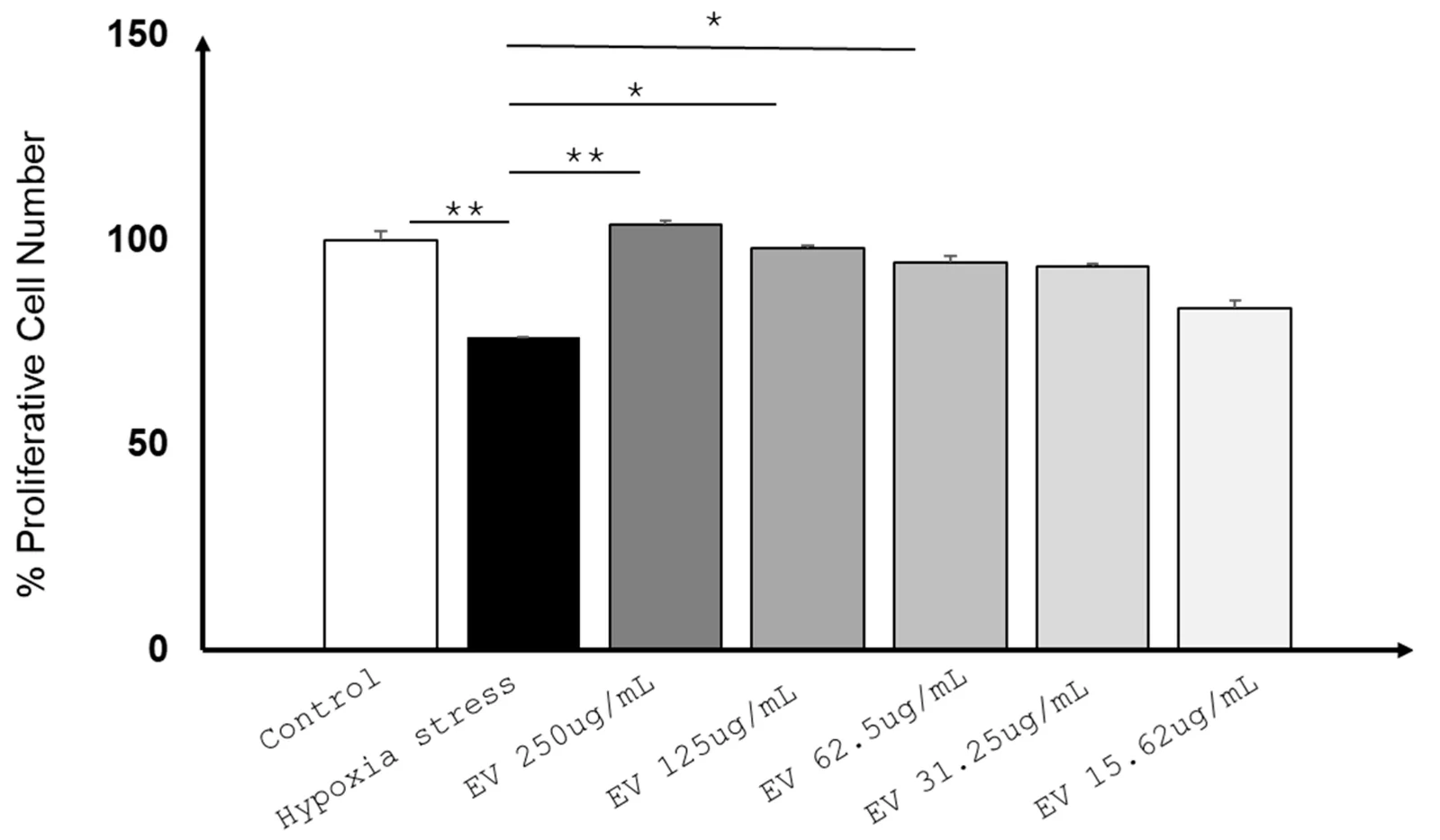
Effect of different EV doses on the MTT response under CoCl_2_-induced chemical hypoxia (*n* = 3), * *p* < 0.05, ** *p* < 0.01.

In the H_2_O_2_ model, the overall difference between groups was significant (*p* < 0.01). The most pronounced improvement compared to H_2_O_2_ stress was observed at a 100 µL EV dose (*p* < 0.001). In the heat stress model, a highly significant difference was observed between groups (*p* < 0.0001); all EV doses evaluated differed from the heat stress condition (*p* < 0.0001). In the chemical hypoxia model, the overall difference was also significant (*p* < 0.01); while the 100, 50, and 25 µL doses provided significant improvement compared to CoCl_2_ stress, the 12.5 and 6.25 µL doses did not. These findings supported the selection of the 100 µL dose for further experiments (Figure 2, Figure 3 and Figure 4). Additionally, morphological images of these cells taken under an inverted microscope also support the MTT results (Appendix A).

### 3.2. EV Treatment Reduced Stress-Induced Apoptosis

**Figure 5:**
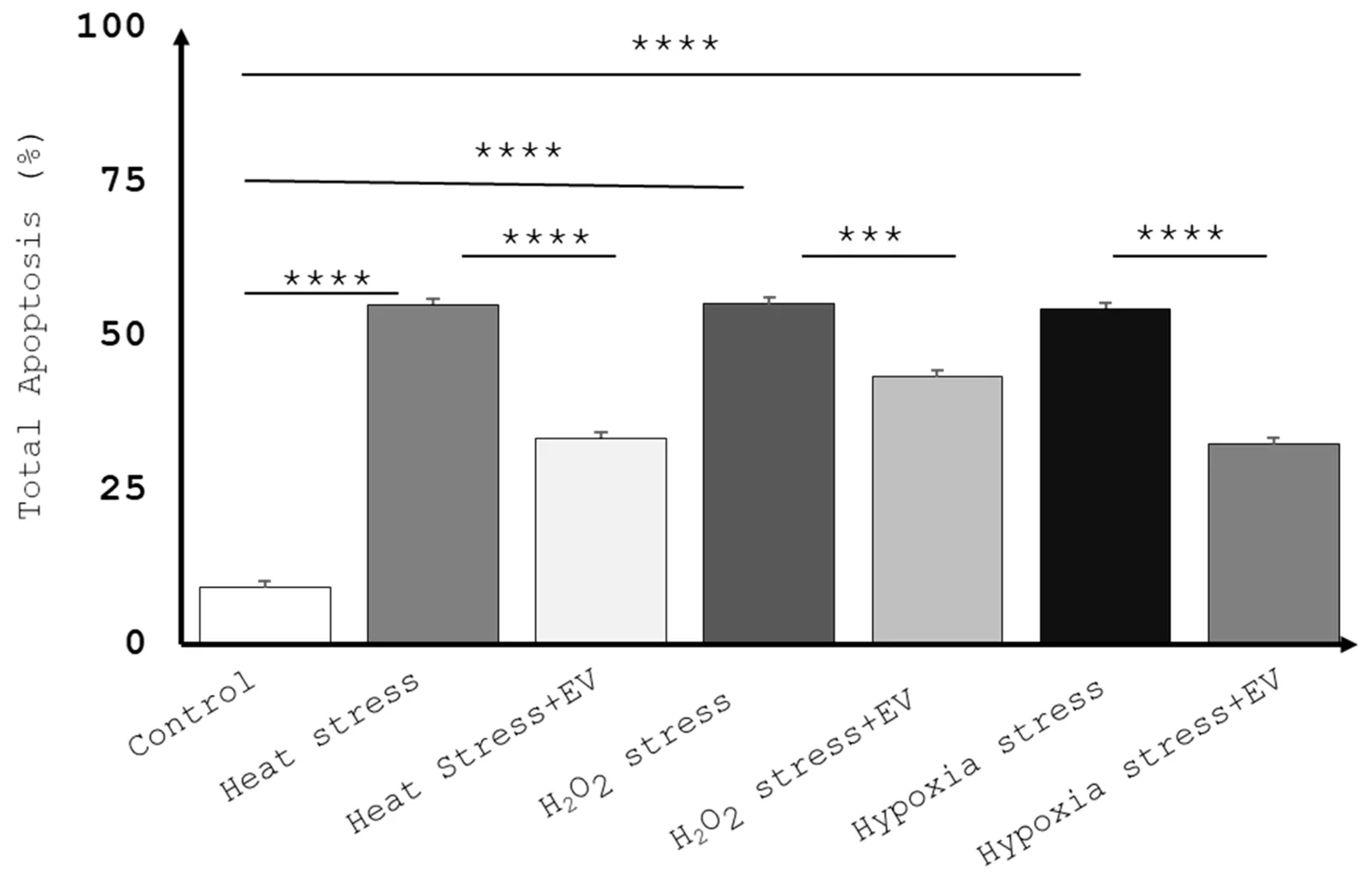
Effect of total apoptosis and EV treatment under chemical hypoxia conditions induced by heat, H_2_O_2_, and CoCl_2_. (*n* = 3), *** *p* < 0.001, **** *p* < 0.0001.

A significant overall difference in total apoptosis was found between the groups (*p* < 0.0001). Total apoptosis was 9.20% in the control group. While heat stress increased apoptosis to 55.06%, EV treatment reduced this value to 33.40% (*p* < 0.0001). Total apoptosis, which was 55.16% under H_2_O_2_ stress, decreased to 43.40% with EV treatment (*p* < 0.0001). Total apoptosis, which was 54.43% under chemical hypoxia, decreased to 32.40% with EV treatment (*p* < 0.0001) (Table 1, Figure 5). Thus, the magnitude of the protective effect was found to be more pronounced in the heat and CoCl_2_ models than in the H_2_O_2_ model.

### 3.3. EV Treatment Suppressed the Intracellular ROS Response

**Figure 6:**
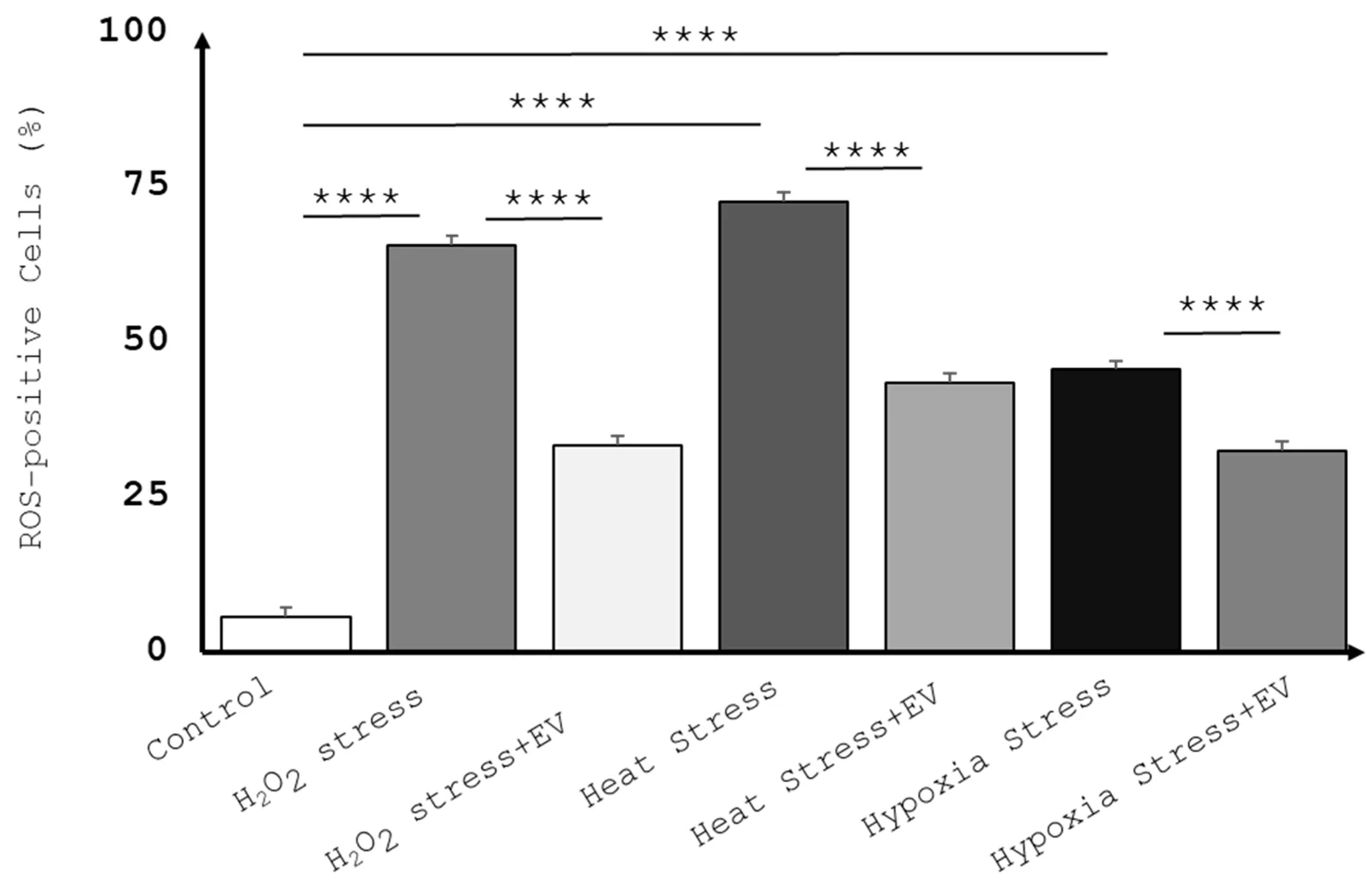
ROS levels in different stress models and the effect of EV treatment (*n* = 3), **** *p* < 0.0001.

Compared to the control, treatment with H_2_O_2_, heat, and CoCl_2_ significantly increased intracellular ROS levels. EV treatment reduced ROS levels in all three stress models compared to the respective stress groups (*p* < 0.0001) (Figure 6). This consistent trend suggests a shared ROS-associated response across the three stress models and indicates that EV treatment attenuates stress-associated ROS accumulation.

### 3.4. EV Treatment Reduced PI Intake

**Figure 7:**
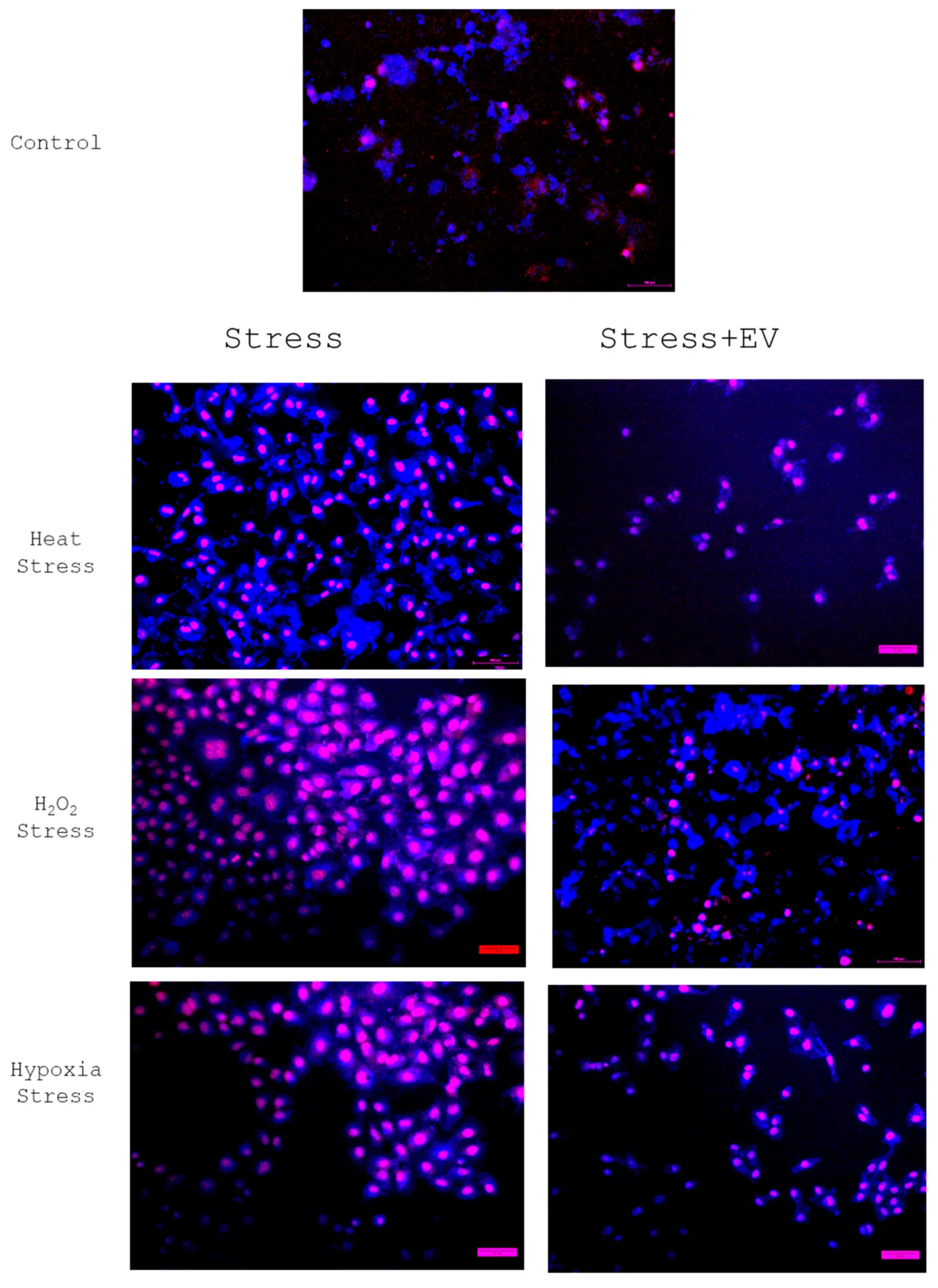

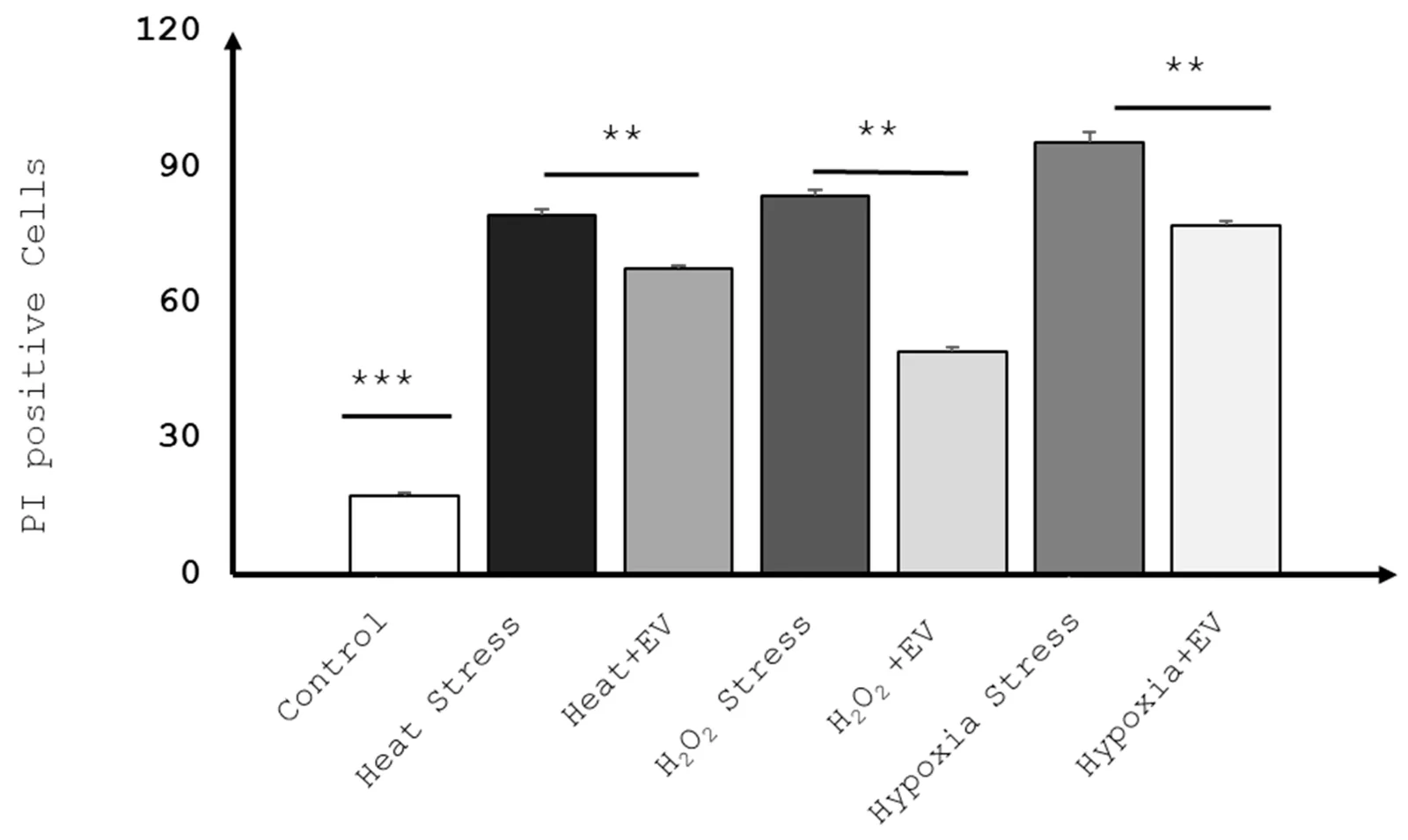
PI values in the Stress and EV groups, ** *p* < 0.01, *** *p* < 0.001. All scale bars represent 100 μm.

PI values decreased from 134.67 to 29.00 under heat stress (*p* < 0.001), from 145.00 to 82.00 under H_2_O_2_ stress (*p* < 0.01), and from 148.00 to 64.00 under chemical hypoxia (*p* < 0.01) (Figure 7). The decrease in PI supported the finding that EV treatment limited the increase in membrane permeability observed in late-apoptotic/necrotic cells (detailed images are attached in Appendix A).

### 3.5. Total RNA Concentration Differed Among Stress Models

**Figure 8:**
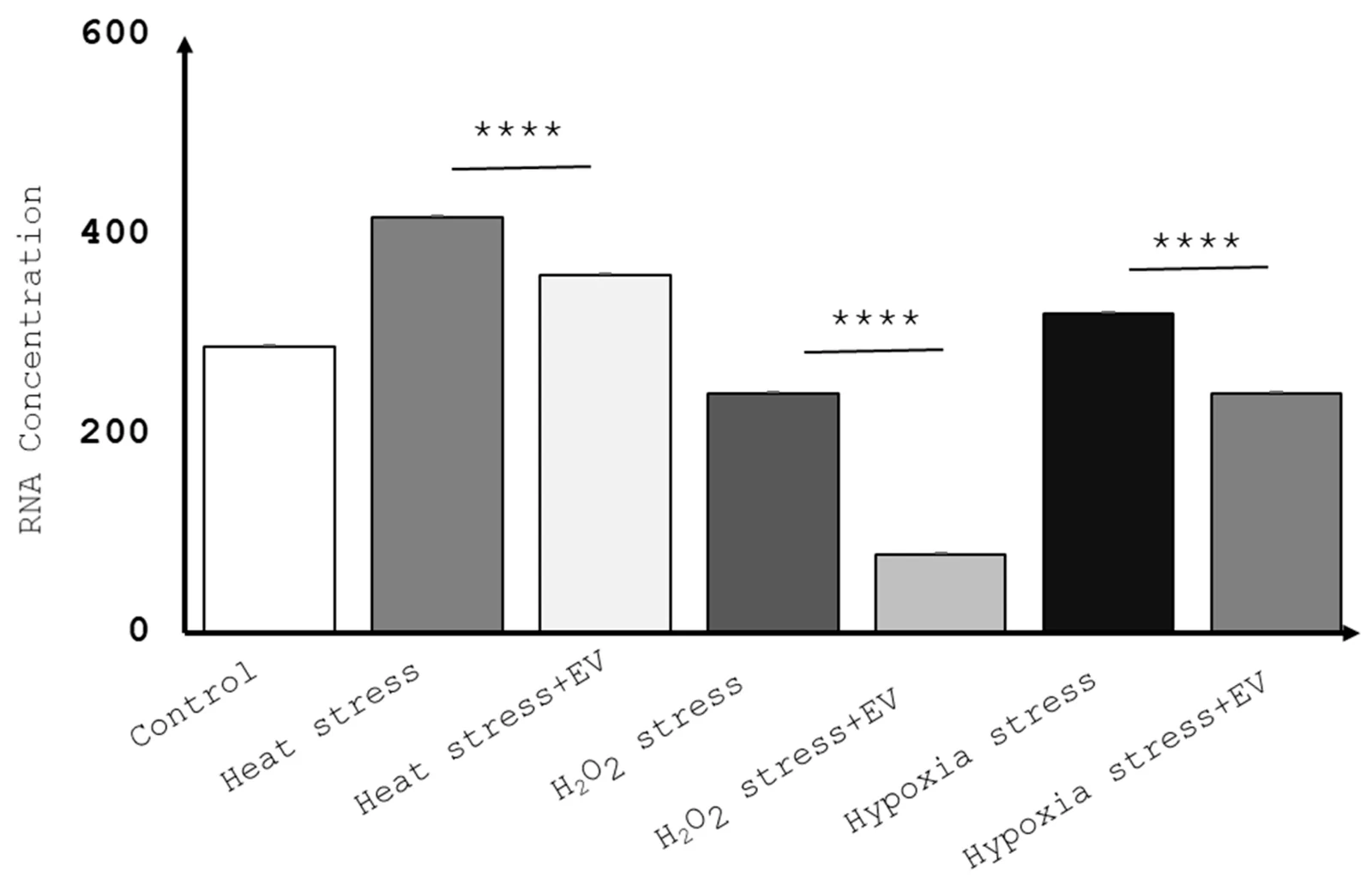
Total RNA concentration (ng/µL) in the stress and EV groups. Data are mean ± SD, *n* = 3; one-way ANOVA and Holm-corrected pairwise comparisons; **** *p* < 0.0001.

RNA concentration differed significantly among the groups (*p* < 0.0001) (Figure 8). With EV treatment, RNA concentration was 58 ng/µL lower than under heat stress, 162 ng/µL lower than under H_2_O_2_ stress, and 80 ng/µL lower than under chemical hypoxia (*p* < 0.0001).

### 3.6. EV Treatment Reduced Cellular miR-21-5p Expression

**Figure 9:**
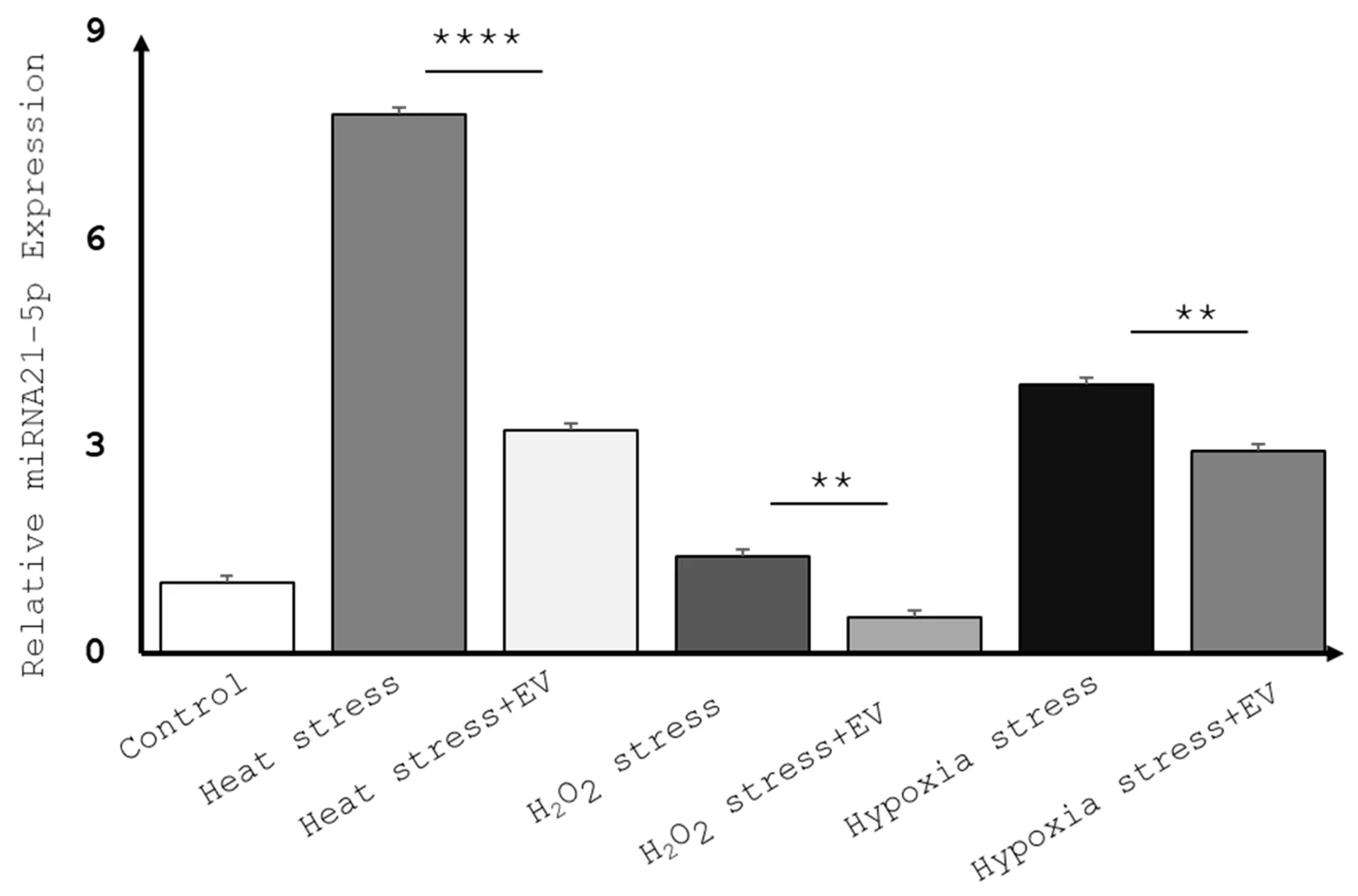
Relative expression of miR-21-5p normalised to U6 in the stress and EV groups (*n* = 3), ** *p* < 0.01, **** *p* < 0.0001.

miR-21-5p expression differed significantly between groups (*p* < 0.0001) (Figure 9). EV treatment reduced miR-21-5p levels by 4.57 relative units compared to heat stress (*p* < 0.0001). In the H_2_O_2_ and chemical hypoxia models, reductions of 0.88 and 0.96 units, respectively, were also observed in the EV groups (*p* < 0.01).

## 4. Discussion

This study demonstrates that EVs derived from human fibroblasts can induce a common protective phenotype in normal lung fibroblasts in response to three distinct stress stimuli. Treatment with heat, H_2_O_2_, and CoCl_2_ all resulted in increased ROS accumulation, PI uptake, and total apoptosis; EV treatment, however, simultaneously reduced all three of these outcomes. The fact that these findings were replicated in the same direction across different stress models suggests that the observed effect may be related to common downstream processes—such as cellular redox balance, membrane integrity, and death signalling—rather than to chemical neutralisation specific to a single stressor.

H_2_O_2_ is an established oxidative stress model capable of inducing increased ROS, decreased glutathione, cell cycle arrest, and cell death in normal pulmonary fibroblasts [1]. However, sensitivity can vary widely depending on the fibroblast source; very different IC_50_ values have been reported for MRC-5 and other primary fibroblasts [4]. The selection of the 40 µM dose used in this study, based on prior dose–response optimization, accounts for this cell-type-specific variability. The reduction in ROS and apoptosis following EV treatment under H_2_O_2_ conditions is consistent with new data indicating that fibroblast EVs limit oxidative damage [11]. However, the fact that the improvement in apoptosis in the H_2_O_2_ model remains more limited than in the heat and CoCl_2_ models suggests that the direct oxidative load may have partially exceeded the compensatory capacity of the EVs.

While CoCl_2_ mimics HIF-1α stabilization by inhibiting prolyl hydroxylase activity, it can also induce ROS production and metal toxicity. Therefore, the effect of CoCl_2_ should not be considered equivalent to physiological hypoxia [5,6]. In our study, the 500 µM CoCl_2_ dose was based on the previously determined IC_50_ in Wl-38 cells and induced a strong ROS/apoptosis response. The fact that EVs reduce total apoptosis in this model indicates that both the oxidative and apoptotic components associated with chemical hypoxia are alleviated together. However, because there was no control for HIF-1α, VEGF, or actual low-O_2_ conditions, it cannot be stated that EVs directly regulate the hypoxia signal; this is a limitation of the study.

The fact that all EV doses improved the MTT response under heat stress and that the 100 µL treatment significantly reduced ROS, PI, and apoptosis suggests that heat-induced proteotoxic stress can also be mitigated by EVs. Although the failure to measure heat shock proteins, components of the unfolded protein response, or mitochondrial membrane potential limits mechanistic differentiation, the concurrent improvement in ROS levels and membrane integrity supports mitochondrial/redox protection as a common candidate mechanism.

It has previously been shown that fibroblast-derived exosomes can reduce inflammation, apoptosis, and DNA damage in UVB-damaged fibroblasts [12]. A study by Yan and colleagues demonstrated that miR-29a-3p in fibroblast exosomes can enhance antioxidant defence by regulating the KEAP1/Nrf2 axis [11]. The present study did not measure Nrf2, KEAP1, or antioxidant enzymes; therefore, it is not possible to propose the same mechanism. Nevertheless, the consistent reduction in ROS across the three stress models justifies future analysis of the exosome cargo in terms of the Nrf2/ARE, SOD, catalase, GPX, and mitochondrial quality control pathways.

The consistency between Annexin V and PI results strengthens the biological integrity of the findings. While Annexin V indicated a reduction in total apoptosis, the decrease in PI suggested that membrane integrity was better preserved. However, PI alone cannot definitively distinguish apoptosis from necrosis; therefore, PI findings should be considered complementary to Annexin V data. In future studies, cell death pathways should be validated using caspase-3/7 activity, PARP cleavage, the BAX/BCL-2 ratio, and mitochondrial membrane potential.

The stress-associated changes in total RNA concentration may reflect broader alterations in cellular RNA content and metabolic/transcriptional activity in response to cellular stress. The reduction observed following EV treatment may be associated with attenuation of this stress response; however, total RNA measurements alone cannot identify the specific RNA species or molecular mechanisms responsible for these changes.

miR-21-5p was selected as a candidate regulatory miRNA due to its association with oxidative stress, apoptosis, and fibroblast responses. miR-21-5p levels exhibit a binding-dependent pattern. miR-21-5p was elevated in stress groups and decreased in parallel with phenotypic improvement following EV treatment. In contrast, it has been reported that miR-21 can exert anti-apoptotic functions via the PDCD4 or PTEN/Akt axes in different cell models [13,14]. This apparent discrepancy reflects the varying roles of miR-21 across cell types, stress durations, and measured compartments. Our measurement is the total miR-21-5p level in recipient Wl-38 cells; based on these results, it is thought that the compensatory/stress-induced miR-21-5p response has also diminished as a result of EV treatment reducing the stress burden.

The biological effects of fibroblast-derived EVs have been studied particularly in skin repair and photoaging models. It has been reported that human dermal fibroblast exosomes reduce UVB-induced inflammation, apoptosis, cell cycle arrest, and DNA damage; in a more recent study, they were shown to limit oxidative damage via the miR-29a-3p/KEAP1/Nrf2 axis [11,12]. These data suggest that fibroblast-derived vesicles may serve not only as matrix regulators but also as cell-free effectors that support redox homeostasis.

miR-21-5p is a binding-dependent microRNA associated with stress, apoptosis, and fibroblast activation. While it exerts an anti-apoptotic effect via the miR-21/PDCD4 or PTEN/Akt axes in some models, it may contribute to pathological cell activation in fibrotic and tumour contexts [13,14].

Moholkar et al. reported that, in the context of lung cancer, exosomes can deliver small molecules and nucleic acid-based therapeutic cargo to target cells and have the potential to reduce systemic toxicity. In the present study, the observed change in miR-21-5p expression following exosome administration suggests that exosome therapy may influence not only phenotypic outcomes, such as cell viability and oxidative stress, but also intracellular regulatory mechanisms [15].

Although this review focuses on tumour biology, the ability of exosomes to modify the molecular and functional responses of recipient cells provides a biological basis for investigating their potential protective effects in various cellular stress models [15].

According to Chen et al., the accumulation of excessive pro-inflammatory factors can lead to the onset of oxidative stress in lung tissue, which in turn causes the development of acute lung injury (ALI). Many drugs currently available on the market cannot cross the lung blood–air barrier; this results in low therapeutic efficacy, which is the primary challenge in ALI treatment. Studies have shown that EVS can cross the blood–air barrier in the lungs and other biological barriers, thereby enhancing therapeutic effects [16,17].

MSC-Exos has become the treatment of choice for many diseases and is a safe and effective, stem-cell-free alternative therapy [17,18]. Exosomes are more stable and more easily modified than mesenchymal stem cells and do not carry a risk of tumour formation. Thanks to their nanometer-scale size and lipid bilayer structure, exosomes can easily cross biological barriers and reach target organs [19]. The therapeutic effect of MSC-Exos has been validated in various diseases, including lung injury, myocardial injury, kidney injury, nerve injury, skin injury, and ageing [17]. Treatments based on MSC-EVs or MSC-Exos have great application potential in the treatment of lung injury associated with oxidative stress. It has been shown that MSC-EVs support the repair of lung injury in the context of ALI by delivering miR-125, miR-181b, and miR-126 [16]. In summary, MSC-EVs play a significant therapeutic role in immune regulation and in reducing oxidative stress in a mouse model of ALI [20]. These findings indicate that MSC-EVs and MSC-exosomes—particularly the miRNAs present in exosomes—may enhance cell regeneration and inhibit apoptosis in oxidative stress-induced lung injury [17,21].

Extracellular vesicles derived from mesenchymal stromal cells (MSCs) are considered promising cell-free biological agents for treating various oxidative stress-related diseases—particularly acute lung injury—owing to their antioxidant, anti-inflammatory, and immunomodulatory properties. Indeed, it has been shown that small extracellular vesicles derived from MSCs reduce oxidative stress and inflammation, improve pulmonary microvascular barrier integrity, and limit damage to lung tissue. However, not only MSCs but also fibroblasts—which play an active role in tissue homeostasis and repair—hold significant potential as therapeutic sources of EVs. Fibroblast-derived EVs can contribute to intercellular communication, extracellular matrix regulation, and the control of the stress response by transferring biological cargo—such as proteins, lipids, and regulatory RNA—to recipient cells [22,23,24].

Furthermore, the ease with which fibroblasts can be cultured and their ability to enable autologous EV production, depending on the cell source, make these cells an accessible source of EVs. Consequently, fibroblast-derived EVs may be useful in reducing cellular stress, similar to the known protective properties of MSC-EVs; however, their effects under different stress conditions have been studied less than those of MSC-EVs. For this reason, investigating the effects of fibroblast-derived small extracellular vesicles on damage caused by heat, oxidative stress, and chemical hypoxia in lung fibroblasts is important for evaluating an alternative cell-free protective approach [25,26,27,28]. Additionally, based on their size distribution and characterisation profile, the isolated EV population appears to be enriched in small EVs (sEVs).

Based on the evidence discussed above, MSC-derived EVs have been extensively investigated as cell-free mediators of regenerative and cytoprotective effects. However, recent evidence shows that fibroblast-derived EVs are also a biologically active, potentially regenerative EV source. Especially, dermal fibroblast-derived EVs have been indicated to modulate inflammatory responses, activated cell proliferation and migration, and contribute to extracellular matrix remodelling and tissue repair. Relative studies have exhibited that EVs derived from fibroblasts possess distinct biological cargo and activities, although some regenerative and anti-inflammatory effects may be more pronounced in MSC-derived EVs. According to this evidence, normal human dermal fibroblasts were selected in the present study as a non-MSC source of EVs to investigate their potential protective activity against stress in lung fibroblasts. Importantly, the present experimental design was not intended to model tissue-specific lung fibroblast-to-lung fibroblast communication, but rather to determine whether EVs derived from normal fibroblasts of a different tissue origin could exert cytoprotective effects across fibroblast types. Nevertheless, EV composition and biological activity are influenced by the tissue origin and physiological state of donor cells; therefore, the dermal origin of the EV-producing fibroblasts should be considered when interpreting the present findings. Direct comparison of dermal fibroblast- and lung fibroblast-derived EVs would be valuable in future studies to determine whether the observed protective effects are donor-tissue dependent [29,30,31].

## 5. Conclusions

EVs derived from human fibroblasts significantly reduced cellular damage caused by heat, H_2_O_2_, and CoCl_2_-induced chemical hypoxia stress in Wl-38 normal lung fibroblasts. The protective effect was demonstrated by increased metabolic activity, decreased ROS production, preserved membrane integrity, and suppressed apoptosis. The reduction in miR-21-5p expression following EV treatment, compared with the stress groups, suggests that this microRNA may serve as a marker of a stress-related response. The findings support the notion that fibroblast-derived EVs exhibit a shared cytoprotective potential against various forms of cellular stress and lay the groundwork for further mechanistic studies into the regulation of lung fibroblast homeostasis via cell-free vesicles.

## Figures and Tables

**Table 1:** Total apoptosis rates in control and stress groups following EV treatment.

Group	Total Apoptosis (%)
Control	9.20 ± 0.61
Heat stress	55.06 ± 3.18
Heat + EV	33.40 ± 0.99
H_2_O_2_ stress	55.16 ± 2.26
H_2_O_2_ stress + EV	43.40 ± 1.57
Hypoxia stress	54.43 ± 3.97
Hypoxia + EV	32.40 ± 3.05

**Figure A1:**
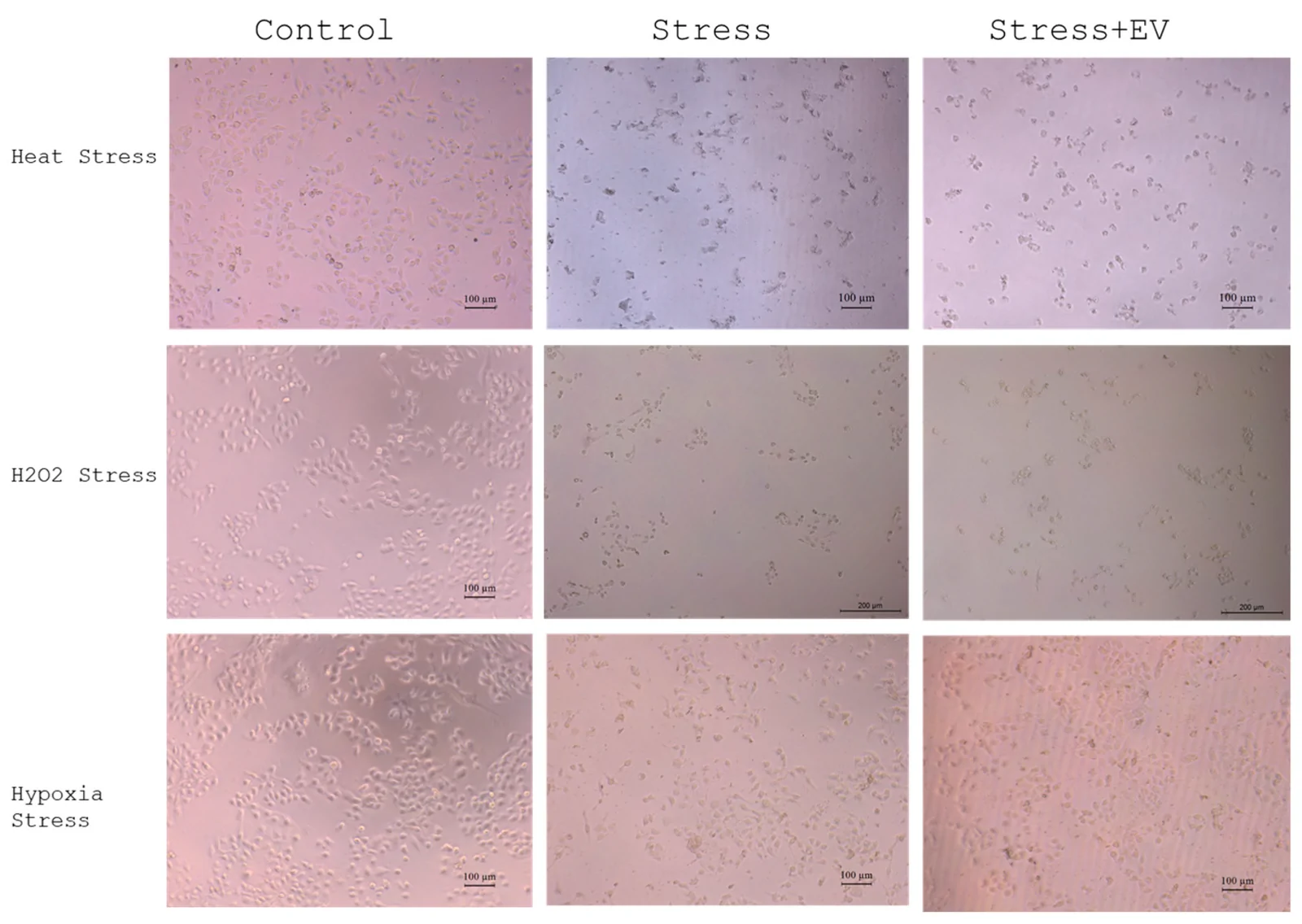
Images of the experimental groups under an inverted microscope.

**Figure A2:**
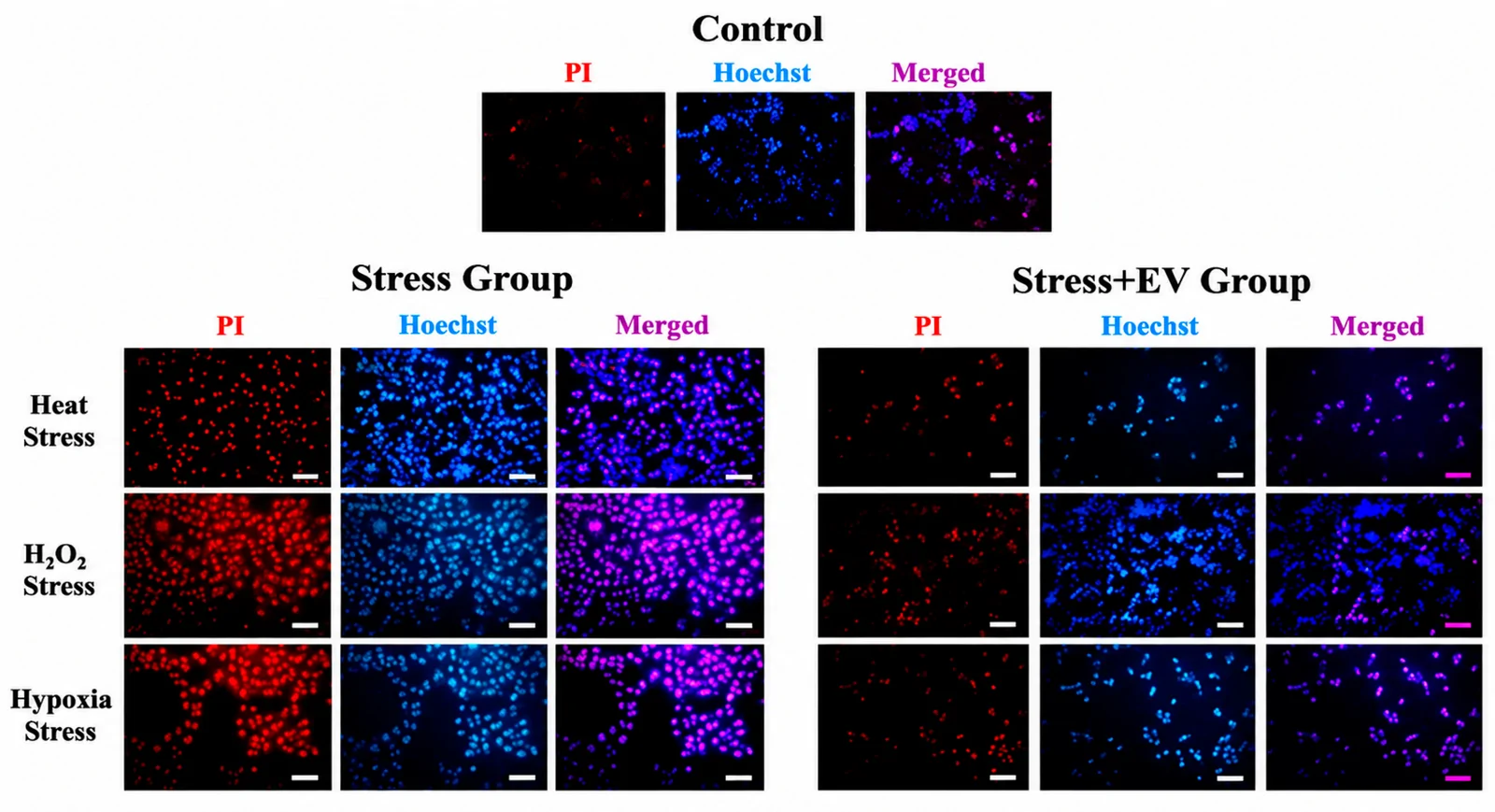
Fluorescent microscopy images of PI and Hoechst staining.

## Data Availability

Data supporting the findings presented in this study are available from the corresponding author upon reasonable request.

## Abbreviations

The following abbreviations are used in this manuscript:

EV	Extracellular vesicles
ROS	Reactive oxygen species
PI	Propodium iodade
NTA	Nanoparticle analysis
SEM	Scanning electron microscope
